# MIC as an Appropriate Method to Construct the Brain Functional Network

**DOI:** 10.1155/2015/825136

**Published:** 2015-02-01

**Authors:** Ziqing Zhang, Shu Sun, Ming Yi, Xia Wu, Yiming Ding

**Affiliations:** ^1^Key Laboratory of Magnetic Resonance in Biological Systems, Wuhan Institute of Physics and Mathematics, Chinese Academy of Sciences, Wuhan 430071, China; ^2^University of Chinese Academy of Sciences, Beijing 100049, China; ^3^School of Mathematics and Statistics, Wuhan University, Wuhan 430072, China; ^4^College of Information Science and Technology, Beijing Normal University, Beijing 100875, China; ^5^State Key Laboratories of Transducer Technology, Shanghai Institute of Technical Physics, Chinese Academy of Sciences, Shanghai 200083, China

## Abstract

Using an effective method to measure the brain functional connectivity is an important step to study the brain functional network. The main methods for constructing an undirected brain functional network include correlation coefficient (CF), partial correlation coefficient (PCF), mutual information (MI), wavelet correlation coefficient (WCF), and coherence (CH). In this paper we demonstrate that the maximal information coefficient (MIC) proposed by Reshef et al. is relevant to constructing a brain functional network because it performs best in the comprehensive comparisons in consistency and robustness. Our work can be used to validate the possible new functional connection measures.

## 1. Introduction

Functional connectivity between brain areas is a hotspot in the field of cognitive neuroscience. In brain functional networks, connectivity is often measured using some form of statistical correlation. Brain activity in one region is correlated with activity in another region to quantify the strength and identify statistical dependency. When repeated for every possible pair of regions, the result is a network characterization of the brain's connectivity, in which brain regions represent network nodes and correlation strengths correspond to connection weights [1, 2].

To date, the main methods of measuring brain functional connectivity include correlation coefficient (CF), partial correlation coefficient (PCF), mutual information (MI), wavelet correlation coefficient (WCF), and coherence (CH). Correlation analysis is the simplest method for analyzing brain functional connectivity; and it is widely used [3–5]. Zalesky et al. [1] recommended using this method to construct a brain functional network. However, contaminations from noise, such as cardiac and blood vessel activities in the brain, could also lead to high correlations [6], and the correlation coefficient only measures linear dependence [7]. Similar to many commonly used statistics, a correlation coefficient is not robust; its value may be misleading if outliers are present [8, 9]. Partial correlation refers to the normalized correlation between two time series after each series has been adjusted by regressing out all other time series of network nodes. One attractive feature of this method is that it attempts to distinguish direct from indirect connections [10]. Marrelec et al. [11] advocated partial correlation as a suitable method to construct a brain functional network. Some researchers have used this method to study the default mode network or the difference between patients and controls [12–15]. Partial correlation is a type of conditional correlation; it still cannot measure a nonlinear association [16, 17]. Coherence is the spectral representation of correlation in the frequency domain and was proposed by Sun et al. [18]. The expression of correlation in the frequency domain enables researchers to study the time course relationship in a natural and intrinsic manner [19]. This method has recently been used by Chang and Glover [20] to investigate nonstationary effects in resting functional magnetic resonance imaging (fMRI) data. However, coherence provides vague information on the actual cortical areas involved because of the complex relationship between the active brain areas and sensor recordings [21]. Wavelet correlation refers to the correlation between wavelet coefficients, which can be obtained from a discrete wavelet transform (DWT) of the time series. Bullmore et al. [22] studied wavelets and the statistical analysis of fMRI of the human brain. Skidmore et al. [23] used wavelet correlation to construct a brain functional network and identified the differences between healthy subjects and subjects with Parkinson's disease. Mutual information (MI), a method in information theory, quantifies the shared information between two variables and can reflect both linear and nonlinear dependencies [24]. This method is popular for measuring the brain functional connectivity [25–27].

In 2011, Reshef et al. [28] proposed a new measure named maximal information coefficient (MIC), which can capture both linear and nonlinear association between two variables. Because of its outstanding performance in measuring different kinds of dependences, it is considered the correlation for the 21st century [29]. Reshef et al. [30] noted that MIC is more equitable compared with natural alternatives, such as mutual information estimation and distance correlation. Since it was proposed, it has been widely used [31–36]. Su et al. [37] were the first to use MIC to construct a brain functional network. However, they only demonstrated that the brain functional connectivity between healthy subjects and schizophrenia subjects calculated using MIC is suitable for classification. As a novel method applied in cognitive neuroscience, there is no explanation accounting for the rationality or advantages of MIC.

The paper demonstrates that MIC is a suitable method to measure brain functional connectivity and illustrates its advantages in constructing a brain functional connectivity network. Thirteen healthy subjects with minimal differences were selected from 75 healthy subjects, and the brain functional network was constructed using MIC, as well as other methods (CF, PCF, MI, WAF, and CH). Based on the node importance, we compared the consistency and robustness of the methods from different aspects of the network. Compared with other methods, MIC performed better in terms of consistency and robustness. Although there are many measures provided to capture the functional connections between brain areas, there is no work to compare the performance of the measures. Our work can be used to validate the possible new functional connection measures.

## 2. Materials and Methods

### 2.1. Subject Information and Data Preprocessing

The data utilized for this paper is available for download at http://fcon_1000.projects.nitrc.org/indi/retro/cobre.html. The study comprised 75 healthy samples (ages ranged from 18 to 65 years in each group). To control external variables for our research, we carefully selected 13 samples with the smallest differences. The ID of samples are 40020, 40051, 40056, 40065, 40091, 40093, 40104, 40114, 40115, 40120, 40127, 40128, and 40129. All subjects were male and right handed according to the Edinburgh Handedness Inventory. Mean age was 24.6 ± 2.6 for the samples. Detailed subject information is provided in Table 1.

All fMRI data were processed using SPM8 (http://www.fil.ion.ucl.ac.uk/spm/) and DPARSF-V2.0 (http://www.restfmri.net/forum/index.php) [38]. For each subject, we removed the first 10 volume images from the RS-fMRI data for scanner stabilization and subject adaptation to the environment, which left 140 volumes for further analysis. Then, we performed slice timing to correct for the acquisition time delay between slices within the same TR; realignment to the first volume to correct inter-TR head motions was performed, followed by spatial normalization to a standard MNI template and resampling to a voxel size of 3 × 3 × 3 mm^3^. No spatial smoothing was applied based on methods from previous studies [39–41]. Finally, we performed bandpass filtering for each voxel in the frequency of 0:01–0:08 Hz to reduce low-frequency drift and high-frequency physiological noise. The RS-fMRI data for each subject were checked for head motion. No subject was excluded according to the criteria that the translation and rotation of head motion in any direction were not more than 1.5 mm or 1.5°. To obtain signals for each region, we applied an automated anatomical labeling (AAL) atlas [42] to parcellate the brain into 90 regions of interest (ROIs) (45 per hemisphere). The names of the ROIs and their corresponding abbreviations are listed in Table 2. The time series for each ROI was calculated by averaging the signals of all voxels within that region.

### 2.2. Maximal Information Coefficient

The MIC, introduced by Reshef et al. [28] in 2011, was used as a measure of association between two random variables *X* and *Y*. The MIC can capture wide range of relationships. The MIC(*X*, *Y*) is the mutual information *I*(*X*, *Y*) between random variables *X* and *Y* normalized by the minimum entropy min⁡{*H*(*X*), *H*(*Y*)} of *X* and *Y*, which can be written as

(1) MICX,Y =IX,Ymin⁡⁡HX,HY=H(X)+HY−HXYmin⁡⁡HX,HY =HY−HY ∣ Xmin⁡⁡HX,HY=H(X)−HX ∣ Ymin⁡⁡HX,HY,

where *H*(*Y*∣*X*) is the conditional entropy, which is the amount of information needed to describe the outcome of *Y* given that the value of *X* is known. For a pair of variables *X* and *Y*, 0 ≤ MIC(*X*, *Y*) ≤ 1 and MIC(*X*, *Y*) = 0 if and only if *X* and *Y* are independent. The MIC is robust to outliers because the estimations of Shannon entropy and conditional entropy are robust [36, 43].

### 2.3. Construct Network

**Figure 1:**
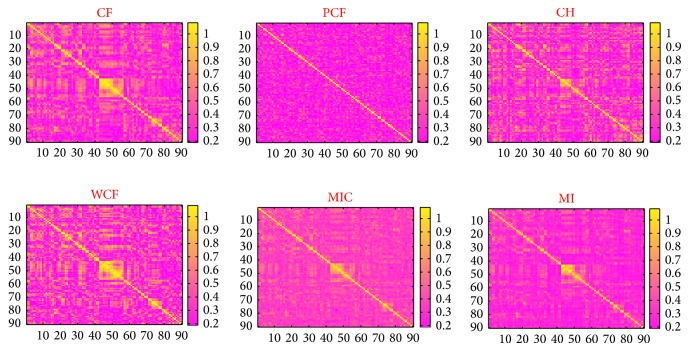
The connection matrices (90 × 90) of a randomly chosen subject from 13 samples were calculated by the 6 previously described methods (CF, PCF, CH, WCF, MIC, MI). The horizontal and vertical axes are the 90 ROIs. The color intensity represents the connection strength. One can get an intuitive understanding of the brain functional connectivity. These connection matrices are the basis for our subsequent research.

For each subject, we obtained a 90 × 90 dependence matrix by calculating the connection strength using one of the 6 previously described methods (CF, PCF, MIC, MI, WCF, and CH) between all ROI pairs. One can check the details about the definitions and computations of the methods in the references.  Figure 1 is the visualization of the six dependence matrices of a randomly selected sample. The correlation matrix was thresholded into a binary matrix. By taking each ROI as a node and the functional connectivity as an edge, we obtained a 90 × 90 adjacency matrix for each subject. The adjacency matrix can be defined as

(2) aij=1if  zij≥T0otherwise,

where *Z* = (*z*_*ij*_) is the connection matrix, *T* is the threshold value, and *A* = (*a*_*ij*_) is the adjacency matrix under threshold *T*. In other words, if the absolute value of *z*_*ij*_ between a pair of brain regions *i* and *j* exceeds a given threshold *T*, an edge is constructed to connect the brain regions; otherwise, there is no edge between them. We know that *T* is an external variable which determines the size of the network. If *T* is too large, we will obtain a network with fewer edges, which may lead to a disconnected network. If *T* is too small, some of the connection strength may be too weak to be significant. To balance these two aspects, 1200 edges were selected as a representative network size. We also have obtained results on different network sizes, and the results were nonsensitive to network size.

### 2.4. Comparison Scheme

We try to conduct both intermethod and intersample comparisons to validate the consistency and robustness. The important results, say key nodes, obtained by a good method should not conflict with that obtained by known methods. So we conduct intermethod comparisons to see which method is more consistent with other methods. Such an aspect we compare is called consistency. Furthermore, a good method should not be sensitive to its operation objects. With 13 subjects of similar physical condition, a good method should obtain similar network properties. This approach represents a type of repeated trials; that is, a good method should always be good, regardless of the experimental targets. Such an aspect we compare is called robustness.

#### 2.4.1. Node Importance

We use two criterions to evaluate the relative importance of a node in a network [43]. One criterion is the degree centrality (DC), which is proportional to the degree of the node. Another criterion is Shannon-Parry centrality (SPC). The former is a popular method. The latter is based on the Shannon-Parry measure of a network and the relative importance of the *i*th node is proportional to *u*(*i*)∗*v*(*i*), where *U* = (*u*(1), *u*(2),…, *u*(*n*)) and *V* = (*v*(1), *v*(2),…, *v*(*n*)) are the left and right eigenvectors of the adjacency matrix *A* of the network. The SPC can effectively illustrate the node importance by synthesizing the node properties and network topology structure [43, 44].

#### 2.4.2. Comparisons on Consistency and Robustness

**Figure 2:**
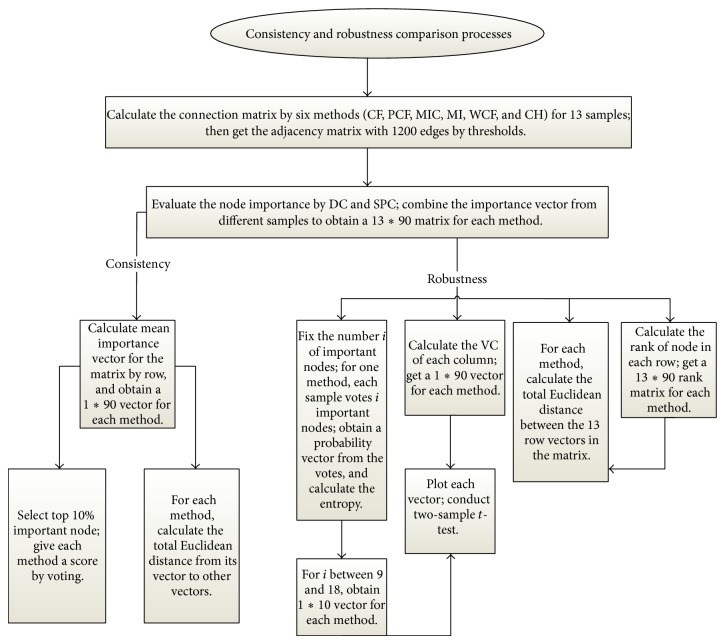
The flowchart summarizes the process of our comparison scheme on two aspects: consistency and robustness. Looking from the top to the bottom following the arrow and the branches, we can have a clear vision of our steps and the steps can be easily realized. We will conduct ten comparisons, four for consistency and six for robustness. To make it clear, “voting” is a metaphor. For instance, when we use 6 methods to extract the top 9 important nodes, we think that the 6 methods are “holding” a vote for their top 9 important nodes from the 90 nodes. The same goes for our samples. In the left branch of consistency part, the six methods have their own important nodes sets after “voting,” but the sets are different; that is to say, the six methods have different “opinions” on important nodes. We give each method a score, respectively, to decide which method's “opinion” is the best. One method's score is the total votes it receives from other methods, including itself. The method which acquires the most votes pools other methods' “opinions” together and is considered more reliable. In the first left branch of the robustness part, the 13 samples “vote” *i*  (9 ≤ *i* ≤ 18) important nodes from the 90 nodes. After “voting,” the 90 nodes have their voting numbers and voting rates (voting number/sum of vote). We can calculate the entropy according to the probability distribution induced by the voting rates. If the entropy is small, it means that the 13 samples have consensus on important nodes, and the corresponding method is more robust.

We conduct the comparisons of the consistency and robustness of the six methods by the flowchart in Figure 2.

## 3. Results

### 3.1. Consistency

We compared the consistency from top 10% important nodes (A) and the total Euclidean distance between importance vectors (B). Since there were two importance criterions DC and SPC, there were four comparisons. The computational results are listed in Tables 3–5.

Table 3 shows the comparison of important nodes; for MIC, the important nodes defined by DC included MEG. L, MEG. R, ROL. L, SFGmed. L, SFGmed. R, INS. L, INS. R, PoCG. R, STG. L, and STG. R. When the node importance was defined by the SPC, nearly the same important nodes were obtained (STG. R instead of SMG. R). MIC had the best rank if the node importance criterion was SPC and ranked the third if SPC was replaced by DC. In the latter case, the score of MIC (33) was very close to the highest score 34.5.

The comparison of the total Euclidean distances of all nodes is shown in Table 4; MIC had the best rank if the node importance criterion is SPC and ranked the second if SPC was replaced by DC. In the latter case, the total distance of MIC was 127.84, which was almost the same as the smallest total distance 126.4.

Table 5 collects the ranking information of the comparisons of consistency.

### 3.2. Robustness

**Figure 3:**
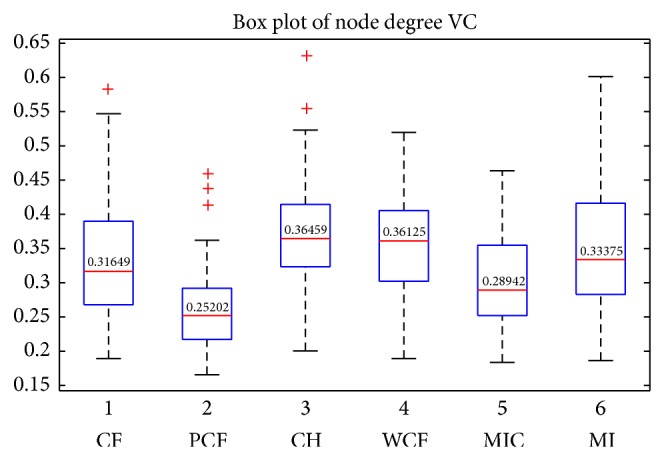
We used degree centrality (DC) to measure node importance and calculated VC of 90 nodes' degree as to 13 samples. Thus, a 1 × 90 vector was obtained for each method. Box plots of these vectors were constructed to determine which method had a smaller average value. When the average VC was smaller, the method was considered to perform better in terms of robustness. The box plot shows the robustness rankings of PCF, MIC, CF, MI, WCF, and CH. Comparisons regarding this aspect indicated that MIC had a good performance. For statistical tests, see Table 8.

**Figure 4:**
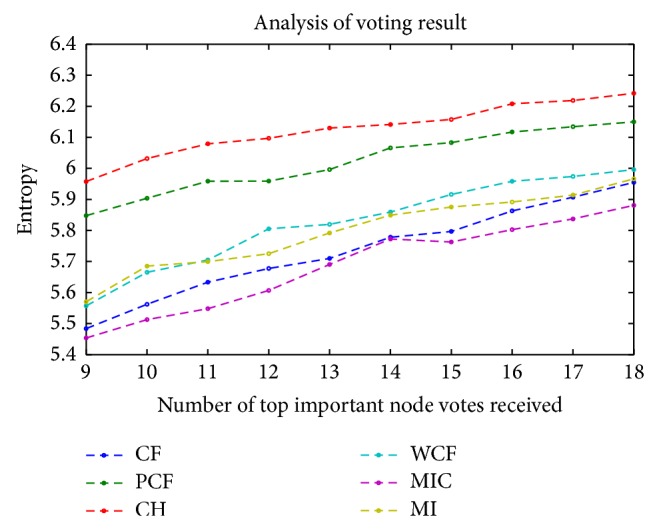
The horizontal axis is the number of important nodes for every subject according to the Shannon-Parry centrality (SPC). The vertical axis is the entropy as a metric to measure results; smaller entropy indicates better performance. Performance results were compared, and MIC performed best. For statistical tests, see Table 9.

Since robustness is an important feature of a method, we conducted six comparisons to examine the robustness of the methods. We calculated the total Euclidean distances between the importance vectors (C) and the importance ranking vectors (D) of the 13 samples. We had four comparisons since there were two importance criterions. On the other hand, we calculated the VC (variation coefficient) of the degrees of the 90 nodes, as well as the voting entropy of all nodes. The results are listed in Tables 6–10 and Figures 3 and 4.

As shown in Tables 6 and 7, in the comparisons of the total distance of all nodes, MIC was ranked the second. In the comparison of the distance between the importance ranking vectors, MIC was ranked first. The box plot (Figure 3) shows the average VC of all nodes, MIC performed the second, and PCF was ranked the first. The results were verified by the two-sample* t*-tests under the confidence level of 95%. In the comparison of the voting entropy of nodes between the 13 samples, MIC performed the best when the top 9–18 (10–20%) nodes were regarded as the important nodes, as shown in Figure 4. MIC passed all the two-sample* t*-tests under the confidence level of 95%. The ranking information is listed in Table 10. Overall, for the robustness comparisons, MIC was consistently ranked in the top two methods.

## 4. Discussions

We compared MIC to five existing methods comprehensively from consistency and robustness. According to the results in Tables 3–5, MIC, CF, and MI are more consistent than WCF, CH, and PCF. The consistency scores of MIC, CF, and MI are very close. Combine the two comparisons in important nodes with criterions DC and SPC in Table 3, the total scores of MIC, CF, and MI are 65, 64.5, and 64.5, respectively. So MIC is more consistent than CF and MI. For the comparisons based on total distance, one can see from Table 4 that MIC also is more consistent than CF and MI. From the four comparisons in consistency, MIC was ranked 3, 1, 2, and 1, CF was ranked 1, 3, 1, and 3, and MI was ranked 2, 2, 3, and 2. The sum of the ranks is 7, 8, and 9 for MIC, CF, and MI, respectively. We conclude that MIC was more consistent than CF and MI because the sum of the ranks is smaller.

For the robustness comparison, we compare six aspects. MIC was ranked the first and PCF was ranked the fifth in the comparisons of the total Euclidean distance between the importance (DC, SPC) ranking vectors and the voting entropy. In the remaining three comparisons, MIC was ranked the second, while PCF was ranked the first. PCF is very robust in the comparisons of total Euclidean distances between the importance (DC, SPC) vectors and variation coefficients of degrees. But it does not mean it is a good method because it performs the worst in all of the consistency comparisons. In fact, it regresses out all the other 88 nodes' influence when calculating the correlation coefficient between a pair of nodes. This leads to relatively uniform results that lack discriminability. We refer to Figure 1 for a typical visualization of a PCF matrix.

## 5. Conclusions

In this paper, we demonstrate that MIC can be used to construct a brain functional network. We compared MIC with five other methods (CF, PCF, MI, WCF, and CH) and ensured that it is suitable for brain functional network construction. In the comprehensive comparisons in consistency and robustness, MIC performs the best, and the results are convincing.

## Figures and Tables

**Table 1:** Sample information.

	*N*	Age range (mean age)	Sex	Right handed

Healthy samples	13	22–27 (24.6)	All male	All

**Table 2:** Names and abbreviations of the ROIs [42].

Regions	Abbr.	Regions	Abbr.
Superior frontal gyrus, dorsolateral	SFGdor	Superior parietal gyrus	SPG
Superior frontal gyrus, orbital	SFGorb	Paracentral lobule	PCL
Superior frontal gyrus, medial	SFGmed	Postcentral gyrus	PoCG
Superior frontal gyrus, medial orbital	SFGmorb	Inferior parietal gyrus	IPG
Middle frontal gyrus	MFG	Supramarginal gyrus	SMG
Middle frontal gyrus, orbital	MFGorb	Angular gyrus	ANG
Inferior frontal gyrus, opercular	IFGoper	Precuneus	PCNU
Inferior frontal gyrus, triangular	IFGtri	Posterior cingulate gyrus	PCC
Inferior frontal gyrus, orbital	IFGorb		
Gyrus rectus	REG	Insula	INS
Anterior cingulate gyrus	ACC	Thalamus	THA
Olfactory cortex	OLF		
		Superior temporal gyrus	STG
Precentral gyrus	PreCG	Superior temporal gyrus, temporal pole	STGp
Supplementary motor area	SMA	Middle temporal gyrus	MTG
Rolandic operculum	ROL	Middle temporal gyrus, temporal pole	MTGp
Median and paracingulate gyrus	MCC	Inferior temporal gyrus	ITG
		Calcarine fissure and surrounding cortex	CAL
Heschl gyrus	HES	Hippocampus	HIP
Cuneus	CUN	Parahippocampal gyrus	PHIP
Lingual gyrus	LING	Amygdala	AMYG
Superior occipital gyrus	SOG		
Middle occipital gyrus	MOG	Caudate nucleus	CAU
Inferior occipital gyrus	IOG	Lenticular nucleus, putamen	PUT
Fusiform gyrus	FG	Lenticular nucleus, pallidum	PAL

**(a):** 

	CF	PCF	MIC	MI	WCF	CH
	MFG. L	ORBsup. L	STG. L	MFG. L	SMG. R	PreCG. L
	MFG. R	MFG. R	MFG. R	ROL. L	ORBmid. L	SFGdor. L
	ROL. L	ORBmid. L	ROL. L	SFGmed. L	ROL. L	MFG. L
	SFGmed. L	CUN. R	SFGmed. L	SFGmed. R	STG. R	MFG. R
	SFGmed. R	PCUN. L	SFGmed. R	INS. L	SFGmed. L	ROL. L
	INS. L	TPOsup. R	PoCG. R	INS. R	SFGmed. R	SFGmed. R
	INS. R	MTG. L	STG. R	PoCG. R	INS. R	PCG. L
	STG. L	TPOmid. R	ANG. L	STG. L	PoCG. R	FFG. R
	STG. R	ITG. R	MFG. L/SMG. R	STG. R	ROL. R/PreCG. R	STG. L

Score	34.5	13	33	33.5	28.5	25.5

**(b):** 

	CF	PCF	MIC	MI	WCF	CH
	MFG. L	ORBsup. L	MFG. L	MFG. L	PreCG. R	PreCG. L
	ANG. L	MFG. R	MFG. R	ROL. L	ORBmid. L	PreCG. R
	ROL. L	ORBmid. L	ROL. L	SFGmed. L	ROL. L	MFG. L
	SFGmed. L	CUN. R	SFGmed. L	SFGmed. R	ROL. R	MFG. R
	SFGmed. R	PCUN. L	SFGmed. R	SFGdor. L	SFGmed. L	SFGdor. R
	INS. L	TPOsup. R	PoCG. R	INS. R	SFGmed. R	PoCG. L
	INS. R	MTG. L	STG. R	PoCG. R	INS. R	PCG. L
	STG. L	ORBsup. R	ANG. L	STG. L	PoCG. R	FFG. R
	STG. R	ORBinf. L	STG. L	STG. R	STG. R	STG. L

Score	30	12	32	31	27	18

**(a):** 

Method	CF	PCF	MIC	MI	WCF	CH
CF	0	48.77	13.90	11.10	22.00	30.70
PCF	48.77	0	47.48	50.36	44.32	40.34
MIC	13.90	47.48	0	14.85	23.80	27.81
MI	11.10	50.36	14.85	0	22.61	31.73
WCF	22.00	44.32	23.80	22.61	0	34.30
CH	30.70	40.34	27.81	31.73	34.30	0

Total	126.4	231.27	127.84	130.65	147.03	164.88

**(b):** 

Method	CF	PCF	MIC	MI	WCF	CH
CF	0	0.0454	0.0117	0.0094	0.0191	0.0299
PCF	0.0454	0	0.0425	0.0442	0.0413	0.0332
MIC	0.0117	0.0425	0	0.0115	0.0192	0.0267
MI	0.0094	0.0442	0.0115	0	0.0180	0.0288
WCF	0.0191	0.0413	0.0192	0.0180	0	0.0328
CH	0.0299	0.0332	0.0267	0.0288	0.0328	0

Total	0.1155	0.2066	0.1116	0.1119	0.1304	0.1514

**Table 5:** A summary of the comparison of consistency. A: consistency in the important nodes; B: consistency in the importance of all nodes. The DC column indicated the importance of the node as defined by degree centrality (DC). The SPC column indicated the importance of the node as defined by the Shannon-Parry centrality (SPC). For the consistency comparison in four aspects, MIC had two times of the first ranking, one time of the second ranking, and one time of the third ranking. Overall, MIC had the best performance. Actually, if we pay attention to Tables 3 and 4, we will find that MIC, CF, and MI have a very similar conclusion.

Aspects	DC						SPC					
	CF	PCF	MIC	MI	WCF	CH	CF	PCF	MIC	MI	WCF	CH
A	1	6	3	2	4	5	3	6	1	2	4	5
B	1	6	2	3	4	5	3	6	1	2	4	5

**Table 6:** C: robustness in the importance of all nodes. We used degree centrality (DC) and Shannon-Parry centrality (SPC) to measure the importance of node in network and put the importance vector calculated in the same method from different sample as the row to construct matrix, so each method obtained a 13 ∗ 90 matrix. The value in the table was the sum of Euclidean distance between different row vectors for one method. D: robustness in the ranking of all nodes in importance. We used degree and Parry measure to measure the importance of node in network and put the importance vector calculated in the same method from different sample as the row to construct matrix, so each method obtained a 13 ∗ 90 matrix. Then we got the rank of node in each row, so each method got a 13 ∗ 90 rank matrix. The value in the table was the sum of Euclidean distance between different row vectors from rank matrix of one method. If this sum of distance was smaller, corresponding method had better robustness. In the aspect C, MIC is ranked the second and is only bigger than PCF. In the aspect D, MIC had the smallest sum of distance, so MIC had better robustness than other methods.

Aspects		CF	PCF	MIC	MI	WCF	CH
C	DC	9148.4	7282.8	8344.8	9536.6	9750.4	10119
	SPC	8.30	6.54	7.59	8.08	8.90	9.45

D	DC	24910	26483	24588	24889	25705	26544
	SPC	24669	26574	24435	24677	25600	26680

**Table 7:** C: robustness in importance of all nodes. D: robustness in the ranking of all nodes in importance. The DC column indicated the importance of the node defined by degree centrality (DC). The SPC column indicated the importance of the node defined by the Shannon-Parry centrality (SPC). For the robustness comparison, MIC had the best ranking in D and was ranked second in C. Actually, PCF was very unstable. In aspect D, PCF had poor performance. In the part of discussion, we will proceed to explain this phenomenon. Overall, compared with other methods, MIC had the best performance in robustness.

	Aspects	DC						SPC					
		CF	PCF	MIC	MI	WCF	CH	CF	PCF	MIC	MI	WCF	CH
Robustness	C	3	1	2	4	5	6	4	1	2	3	5	6
	D	3	5	1	2	4	6	3	5	1	2	4	6

**Table 8:** We conducted one side two-sample *t*-test between each two methods under the confidence level of 95%. In this chart we showed the *P* value of the test in which the method's mean in the row was smaller than that in the column. We arranged the methods according to their rank. The events in the symmetric position of the table were complementary, so we just showed the bold font data. We could see PCF passed 5 tests and was ranked first. MIC passed 4 tests and was ranked second.

	PCF	MIC	CF	MI	WCF	CH
PCF		**>0.9999**	**>0.9999**	**>0.9999**	**>0.9999**	**>0.9999**
MIC			**0.9966**	**0.9999**	**>0.9999**	**>0.9999**
CF				**0.8758**	**0.9415**	**0.9936**
MI					**0.5986**	**0.8714**
WCF						**0.8447**
CH						

**Table 9:** We conducted one side two-sample *t*-test between each two methods under the confidence level of 95%. In this chart we showed the *P* value of the test in which the method's mean in the row was smaller than that in the column. We arranged the methods according to their rank. The events in the symmetric position of the table were complementary, so we just showed the bold font data. We could see MIC passed 5 tests, which performed best.

	MIC	CF	MI	WCF	PCF	CH
MIC		**0.7668**	**0.9556**	**0.9757**	**>0.9999**	**>0.9999**
CF			**0.8277**	**0.9011**	**0.9999**	**>0.9999**
MI				**0.6796**	**0.9998**	**>0.9999**
WCF					**0.9984**	**>0.9999**
PCF						**0.9870**
CH						

**Table 10:** In the robustness comparison, MIC was ranked the second in the aspect of VC of degree centrality (DC); MIC had the best ranking in terms of entropy of important nodes. Overall, compared with other methods, MIC performed best in terms of robustness.

Approach	CF	PCF	MIC	MI	WCF	CH
VC of DC	3	1	2	4	5	6
Entropy of important nodes	2	5	1	3	4	6
